# Dietary Intake among Adolescents in a Middle-Income Country: An Outcome from the Malaysian Health and Adolescents Longitudinal Research Team Study (the MyHeARTs Study)

**DOI:** 10.1371/journal.pone.0155447

**Published:** 2016-05-17

**Authors:** Hazreen Abdul Majid, Liyana Ramli, Sim Pei Ying, Tin Tin Su, Muhammad Yazid Jalaludin, Nabilla Al-Sadat Abdul Mohsein

**Affiliations:** 1 Department of Social and Preventive Medicine, Faculty of Medicine, University of Malaya, Kuala Lumpur, Malaysia; 2 Centre for Population Health, Faculty of Medicine, University of Malaya, Kuala Lumpur, Malaysia; 3 Department of Pediatrics, Faculty of Medicine, University of Malaya, Kuala Lumpur, Malaysia; University of Utah, UNITED STATES

## Abstract

Optimal nutrition is essential for healthy growth during adolescence. This study aims to investigate the baseline nutritional intake of Malaysian adolescents by gender, body mass index, and places of residence, both urban and rural. A *cohort study* was conducted consisting of 794 adolescents (aged 13-years) attending 15 public secondary schools from the Central (Kuala Lumpur and Selangor) and Northern (Perak) Regions of Peninsular Malaysia. Qualified dietitians conducted a 7-day historical assessment of habitual food intakes. Facilitated by flipcharts and household measurement tools, detailed information on portion sizes and meal contents were recorded. Nutritionist Pro™ Diet Analysis software was also used to analyze the dietary records.The mean age of the adolescents was 12.86 ± 0.33 y; the mean energy intake was 1659.0 ± 329.6 kcal/d. Males had significantly (*P* < .001) higher energy intake than females (1774.0 ± 369.8 *vs* 1595.2 ± 320.6 kcal/d); adolescents in rural schools consumed more energy and cholesterol (*P* < .001) compared to adolescents in urban schools (1706.1 ± 377.7 kcal/d and 244.1 ± 100.2 mg/d, respectively). Obese adolescents in rural schools consumed more energy and sugar (1987.6 ± 374.0 kcal/d and 48.9 ± 23.0 g/d) (p-value <0.001).The dietary intake of normal weight versus obese adolescents differs by the location of their school. Thus, the implementation of a structured and tailored intervention is recommended to help minimize this nutritional inequality.

## Introduction

Adolescence is defined as a period of human growth and development that occurs after childhood and before adulthood, between the ages of 10 and 19 [1]. To meet the macro and micronutrient requirements of adolescence, optimal nutrition is critical. These requirements are different for males and females [2]. To curb the escalating problem of nutritional extremes in adolescent populations—consuming under and over what is necessary—the World Health Organization (WHO) has recommend a healthy diet [3] of adequate fruits and vegetables. These recommendations have been actively promoted to all age groups, to help minimize the risk of developing heart disease, cancer and other chronic diseases. [3,4].

It has been shown that over-nutrition and poor lifestyle choices can lead to obesity [5]. Despite the complex etiology of obesity, a study reported that many researchers assert that the main cause of obesity is an energy imbalance between calories consumed and calories expended [6]. The WHO has observed a global shift in dietary preferences, towards energy-dense foods that are higher in fats and sugars, yet poorer in vitamins and minerals [3]. In addition, the trend of decreased physical activity level, which is due to the rise of sedentary lifestyles [2], has worsened the obesity problem for adolescent persons.

The prevalence of overweight adolescents in the United States aged 12 to 19 years has increased from 10.5% in 1988–1994 to 15.5% in 1999–2000 [7]. A similar trend has been observed in Malaysia, were the prevalence of overweight adolescents has risen from 9.5% in 1997 to 19.6% in 2007 [8]. Based on the National Health and Morbidity Survey of 2011, the prevalence of obese adolescents aged 10 to 14 years was 6.3% (95% confidence interval: 5.3–7.6) [9]. However, a more recent study has shown that the prevalence of obesity in adolescents aged 13 years was 23.9%, suggesting that almost 1 in 4 adolescents are overweight or obese [10]. This obesogenic environment is prevalent in rural settings. In Iowa [11], the prevalence of overweight persons was higher among rural children (25%) when compared to children from urban areas (19%, P < .001) and small cities (17%, P < .001). Rural children were 1.47 times more likely to be categorized as overweight than children from small cities. These findings in Iowa reflect a wider tread across rural America, where being overweight is more prevalent for rural children (16.5%) than for urban children (14.3%) [12].

For adolescents, dietary guidelines recommend a healthy and balanced variety of food selections, as well as eating in moderation, in order to optimize adolescent nutrition. Despite these guidelines, evidence has shown that dietary habits and intake among adolescents is poor because of binging on energy-dense snacks and drinks [2,13,14]. Focusing on dietary intake, some studies have demonstrated the prevalence of a low-fiber, high-fat diet among adolescents [15–17]. In Malaysia, studies on adolescent dietary intake [17,18] are limited, mainly because the recruited adolescents were only from one State, or from a smaller sample size, which makes it difficult to apply knowledge across the entire country. Thus far, there have been no large cohort studies that followed the dietary intake of adolescents in Malaysia. The aim of the present study was to investigate the adolescent dietary intake of Malaysian adolescents, from the first wave of a large cohort study, and specifically investigating gender, body mass index and places of residence, both urban and rural.

## Material and Methods

### Participants

The MyHeARTs study is a *cohort study* that recruited secondary school students from three States in the Central and Northern Regions of the Peninsular Malaysia, namely Selangor, Federal Territory of Kuala Lumpur, and Perak. Exactly 1361 students were recruited. The schools were stratified into urban and rural, based on criteria provided by the Department of Statistics, Malaysia.The dietary data used in this paper are from the first wave of this cohort, held between March and May, 2012. Details of the study were reported using published protocol [10], and were approved by ethics committees at the University of Malaya Medical Centre (MEC Ref. No: 896.34).

Our study included school children aged 13 years, in their first year at a public secondary school. As a requirement, the participants needed to read Malay, the national language of Malaysia. Other types of schools, like boarding, religious, and vernacular schools, were all excluded from out study, as the majority of students attending these schools encompass a single ethnic group. Participants and their parents or guardians received detailed and written information about the study as well as consent forms. Prior to participation, all participants and their parents or guardians were required to submit a completed consent form, thus indicating their willingness to participate. To categorize the education level of the parents, primary school was defined as education obtained in a school between the ages of 7 and 12 years—this was also called primary education; secondary school was considered as education obtained in a school between the ages of 13 and 17 years—this was also called secondary education; STPM, or a College or University level education, was defined as education obtained following the completion of secondary school—also called tertiary education. Based on family income, the socioeconomic status (SES) of the participants was categorised into low (<RM1500), middle (RM 1501–RM 5000) and high (>RM5000) SES [19].

### Instruments and procedures

#### Dietary assessment

Habitual food and beverage intakes were assessed during a 7-day diet history. Throughout this study, seven qualified and trained dieticians interviewed students and collected their information on food and drink consumption, at breakfast, mid-morning snack, lunch, afternoon tea, dinner, and supper. As the weight of the food (measured in grams) could be not be obtained for specific foods during the interview, flip charts were used from the validated Malaysian Atlas of Food Exchanges and Portion Sizes [20] guidance, which contains information on local foods and average portion sizes. These charts were used as a supplementary tool to assist the study participants during their dietary evaluations, and to help estimate the portion sizes of the foods they consumed. During each interview, the dieticians used visual examples of household measurement tools, like a plate, cup, glass, bowl, ladle, or spoon, to aid with communication. Each diet history was translated into a food summary or sheet, where all food items were summarized, and each with their own code: this summary included the day, the time, the name of food or drink, the quantity of the meal—measured in grams or milliliters—as well as the food code. Nutritional intake was calculated using the Nutritionist Pro™ Diet Analysis software, from Axxya Systems, USA. Newer Malaysian food recipes, as well as newer products that were not listed in the Nutrient Composition of Malaysian Food guidance, were added to the Nutritionist Pro™ database, and thereafter the food summary showed 223 new products and 523 new recipes. When these newer recipes were entered into the database, portion sizes were calculated based on a standard recipe size, i.e. per serving size, and this information was obtained from the website www.kongsiresepi.com; however, the standard measurement of ingredients used to make the recipe was aquired and validated using the Nutrient Composition of Malaysian Food [21] guidance, as well as via the Malaysian Atlas of Food Exchanges and Portion Sizes documentation [20]. Product information was taken directly from food packaging, for each food item.

To determine and correct for underreported dietary intake, a ratio was calculated between energy intake and the predicted basal metabolic rate (EI:BMR). The basal metabolic rate (BMR) was calculated using the Henry and Rees (1991) equations [22], as these were built on a database of mixed populations that included persons from tropical regions, which is similar to the population mix and climate of Malaysia. The EI:BMR ratio was calculated by first dividing the average energy intake with the BMR; then the ratio obtained was compared with the cut-off point from Kersting et. al. [23]; then the values of the ratio that were lower than the cut-off point from Kersting et al. were excluded from data analysis.

#### Physical evaluation

Height was measured, without socks and shoes, using a calibrated vertical stadiometer (Seca Portable 217, Seca, Birmingham, UK) and was recorded to the nearest millimeter, or one-tenth of a centimeter (0.1 cm). Weight was measured with light clothing, using a digital, electronic weighing scale (Seca 813, Seca, Birmingham, UK) and was recorded to the nearest hectogram, or one-tenth of a kilogram (0.1 kg). Body mass index (BMI) was calculated in kilograms, which was then divided by the square of the participant’s height in meters; the result was classified using the International Obesity Task Force (IOTF) standard. Body fat composition was measured using a portable body composition analyzer (Tanita SC-240 MA, Body Composition Analyzer, Tanita Europe B.V., Amsterdam, The Netherlands). Waist and hip circumference were each measured using a non-elastic Seca measuring tape (Seca 201, Seca, Birmingham, UK) to the nearest millimeter, or one-tenth of a centimeter (0.1 cm).

#### Data analysis

A normality test (Kolmogorov-Smirnov test) was applied to all variables in this study. Data for each gender were analyzed separately. In addition, separate analyses were conducted for body mass index and for places of residence, both urban and rural. Quantitative variables were described as frequencies and percentages; if variances were equal when comparing the two groups (dietary intake by gender and places of residence) for the quantitative variable, then a *t-test* was used. During the analysis of variance, a 95% confidence interval was used that was based on the homogeneity of the variances, to compare the dietary intake for each BMI group (normal weight, underweight, overweight, and obese groups). If the result of the Levene’s test was >0.05 this meant that the assumption of equality variances was matched. Associations between adolescent body mass index and other obesity risk factors were tested using binary logistic regression. All statistical analyses were completed using SPSS software for Windows (Version 20.0, Chicago, IL, US). A p-*value* <0.05 was deemed significant.

## Results

Exactly 1361 adolescents were involved in this study. From this total, 1290 adolescents completed a dietary interview. Data from 794 dietary histories were eventually analysed, after excluding those that were underreported (38.4%), which was determined by calculating the ratio of measured energy intake against the predicted basal metabolic rate (EI:BMR) [22,23]. The socio-demographic characteristics of the initial cohort of 794 persons is presented in **Table 1**. The majority of the participants were females 64.4% (*n* = 511), while 35.6% (*n* = 283) were males; regarding places of residence, 50.3% adolescents (*n* = 399) were from urban areas, while the remaining 49.7% (*n* = 395) were from rural areas. The BMI was classified using IOTF standards, which showed that more than half of the adolescents had a normal BMI (57.9%), and that the prevalence of overweight and obese persons among adolescents was 9.4% (n = 75) and 2.9% (n = 23) respectively. Most of the adolescents in this study were ethnically Malay (n = 582, 82%), followed by Indian (n = 63, 8.9%), Chinese (n = 34, 4.8%), and other (n = 31, 4.4%). The percentage of those that reported smoking was 9.2% (n = 65): 5.4% were male (n = 38), 3.8% were female (n = 27). Also, 45.1% of the adolescents self-reported exercising once or twice a week (n = 320), although only 6.2% of the adolescents self-reported daily exercise (n = 44). The majority of the adolescents in this study had a low SES (n = 331, 49.0%). Also, 13.8% of those interviewed had parents with CVD risk factors (n = 86).

**Table 1:** Characteristic of adolescents age 13 y attending secondary school by gender.

Characteristic	Male	Female	Total (%)
	No (%)*n* = 283 (35.6)	No (%)*n* = 511 (64.4)	*n* = 794
Places of residence			
Urban	115 (14.5)	284 (35.8)	399 (50.3)
Rural	168 (21.2)	227 (28.6)	395 (49.8)
Ethnicity			
Malay	195 (27.5)	387 (54.5)	582 (82.0)
Chinese	15 (2.1)	19 (2.7)	34 (4.8)
Indian	17 (2.4)	46 (6.5)	63 (8.9)
Others	12 (1.7)	19 (2.7)	31 (4.4)
Smoking status			
Yes	38 (5.4)	27 (3.8)	65 (9.2)
No	201 (28.3)	444 (62.5)	645 (90.8)
Alcohol intake			
Yes	10 (1.4)	4 (0.6)	14 (2.0)
No	229 (32.3)	467 (65.8)	696 (98.0)
Physical activity (for last 7 days)			
Never	63 (8.9)	156 (22.0)	219 (30.8)
Sometimes (1–2 times last week)	104 (14.6)	216 (30.4)	320 (45.1)
Often (3–4 times last week)	37 (5.2)	62 (8.7)	99 (13.9)
Quite often (5–6 times last week)	15 (2.1)	13 (1.8)	28 (3.9)
Very often (≥ 7 times last week)	20 (2.8)	24 (3.4)	44 (6.2)
Body Mass Index (IOTF standards kg/m²)			
Underweight (< 15.8^m^; < 16.3^f^)	91 (11.5)	145 (18.3)	236 (29.7)
Normal (15.8—< 21.9^m^; 16.3—< 22.6^f^)	164 (20.7)	296 (37.3)	460 (57.9)
Overweight (< 21.9—< 26.8^m^; 22.6—< 27.8^f^)	19 (2.4)	56 (7.1)	75 (9.4)
Obese (≥ 26.8^m^; ≥ 27.8^f^)	9 (1.1)	14 (1.8)	23 (2.9)
Highest education of mother			
Never schooled	5 (0.8)	11 (1.7)	16 (2.4)
Primary education	25 (3.8)	55 (8.3)	80 (12.0)
Secondary education	141 (21.1)	300 (45.0)	441 (66.2)
Tertiary education	46 (6.9)	83 (12.5)	129 (19.4)
Highest education of father			
Never schooled	4 (0.6)	9 (1.4)	13 (2.1)
Primary education	23 (3.6)	51 (8.1)	74 (11.7)
Secondary education	140 (22.2)	275 (43.6)	415 (65.8)
Tertiary education	41 (6.5)	88 (13.9)	129 (20.4)
Socio-Economic Status (SES) of parents			
Low	108 (16.0)	223 (33.0)	331 (49.0)
Middle	93 (13.8)	180 (26.6)	273 (40.4)
High	24 (3.6)	48 (7.1)	72 (10.7)
Parents with CVD risk factors			
Yes	21 (3.4)	65 (10.4)	86 (13.8)
No	165 (26.4)	352 (56.3)	517 (82.7)

The overall nutrient intakes of male and female adolescents is illustrated in **Table 2**. The mean energy intake of adolescents for males was 1774 ± 369 kcal/d, and for females was 1595.2 ± 320.6 kcal/d. Male adolescents consumed significantly more energy, macronutrients and sugar, compared to females—fiber consumption was the exception to this rule. As for micronutrients, female adolescents consumed slightly more vitamin D than males (0.7 μg/d, 28 units/d). **Table 3** outlines the percentages of Recommended Nutrient Intake (RNI, 2005) [24] achieved by the adolescents; it shows that both males and females consumed inadequate intake of energy, vitamin D, and calcium; it also shows that females consumed inadequate levels of iron (less than 100%); and shows that both genders achieved the optimal intake of proteins and fats (more than 100%).

**Table 2:** 7-d nutrient intake for adolescents age 13-y-old attending secondary schools by gender.

Macronutrients	Overall mean (95% CIª)	Male mean (95% CI)	Female mean (95% CI)	*P*-value
	*n* = 794	*n* = 283	*n* = 511	
Energy (kcal/d)	1659.0 (1634.6–1683.3)	1774.0 (1730.8–1817.3)	1595.2 (1567.4–1623.1)	< 0.001
(MJ/d)	6.9 (6.8–7.0)	7.4 (7.2–7.6)	6.7 (6.6–6.8)	
Protein (g/d)	61.2 (60.1–62.2)	65.7 (63.6–67.7)	58.7 (57.4–59.7)	< 0.001
Carbohydrate (g/d)	229.0 (225.3–232.7)	245.2 (238.6–251.8)	220.0 (215.7–224.2)	< 0.001
Crude fiber (g/d)	2.9 (2.8–3.1)	2.9 (2.7–3.1)	3.0 (2.8–3.1)	NS
Sugar (g/d)	34.3 (33.1–35.4)	34.7 (32.5–36.8)	34.1 (32.7–35.5)	NS
Fat (g/d)	55.5 (54.4–56.5)	59.7 (57.6–61.7)	53.2 (52.0–54.3)	< 0.001
Cholesterol (mg/d)	223.3 (216.6–229.9)	248.8 (236.5–261.0)	209.1 (201.6–216.7)	< 0.001
Saturated fatty acid (g/d)	10.9 (10.5–11.2)	12.0 (11.2–12.7)	10.3 (9.9–10.7)	< 0.001
Monounsaturated fatty acid (g/d)	8.3 (8.0–8.5)	9.0 (8.5–9.5)	7.9 (7.6–8.1)	< 0.001
Polyunsaturated fatty acid (g/d)	6.0 (5.9–6.2)	6.4 (6.1–6.7)	5.8 (5.6–6.0)	0.005
**Micronutrients**				
Vitamin D (μg/d)	0.6 (0.5–0.7)	0.4 (0.2–0.6)	0.7 (0.5–0.8)	< 0.001
Sodium (mg/d)	2289.5 (2239.9–2339.1)	2460.0 (2369.3–2550.6)	2195.1 (2138.1–2252.2)	< 0.001
Potassium (mg/d)	1033.2 (1012.3–1054.1)	1096.6 (1059.0–1134.1)	998.1 (973.6–1022.7)	< 0.001
Calcium (mg/d)	377.4 (365.1–389.7)	378.9 (358.6–399.2)	376.5 (361.0–392.1)	NS
Magnesium (mg/d)	114.7 (110.9–118.5)	124.8 (116.7–132.9)	109.1 (105.4–112.8)	0.001
Phosphorus (mg/d)	842.3 (823.0–861.6)	914.6 (878.2–951.0)	802.3 (780.8–823.7)	< 0.001
Iron (mg/d)	14.2 (13.8–14.5)	15.2 (14.6–15.9)	13.6 (13.2–14.0)	< 0.001

CI- Confidence interval

NS—Not significant

**Table 3:** Energy, macronutrient and micronutrient intakes and percentages, of recommended nutrient intake (RNI, 2005) achievements, of the adolescents by gender and places of residence.

	RNI for 13 y		RNI achieved (%) by adolescents			
	Male	Female	Male		Female	
			Urban	Rural	Urban	Rural
Energy (kcal/d)	2690	2180	65.5	66.3	71.2	75.7
(MJ/d)	11.3	9.1				
Protein (g/d)	63	55	102.0	105.7	104.0	110.0
Carbohydrate (%)	55–75% from EI		36.0	36.8	39.1	41.9
Fat (g/d)	57–86	46–69	105.9	103.7	118.0	113.5
Vitamin D (μg/d)	5		12.0	6.0	14.0	14.0
Calcium (mg/d)	1000		40.8	35.9	38.3	36.9
Iron, (mg/d) ª	15		104.0	93.3	89.3	92.0

ª 10%bioavailability

**Table 4** shows the 7-day nutritional intake for adolescents attending secondary schools by body mass index. It shows that obese adolescents consumed significantly more energy (1987.6 ± 374.0 kcal/d) and macronutrients, like proteins (70.5 ± 18.6 g/d), carbohydrates (269.7 ± 53.3 g/d), fats (70.3 ± 15.5 g/d) and sugar (48.9 ± 23.0 g/d), when compared to normal weight adolescents (p-value <0.001). Concerning micronutrients, obese adolescents consumed more sodium (2810.4 ± 923.5 mg/d), phosphorus (1017.8 ± 330.8 mg/d) and iron (17.3 ± 5.2 mg/d), when compared to other groups (p-value <0.001). Furthermore, adolescents from rural schools consumed significantly higher amounts of energy, when compared to adolescents from urban schools (1706.1 ± 377.7 kcal/d vs 1612.3 ± 312.5 kcal/d, *P* <0.01), as shown in **Table 5**. Also, significant differences were observed, in the consumption of carbohydrates, cholesterol, potassium, and phosphorus, when comparing the adolescents of rural and urban schools (*P* < 0.01). Separate analysis was done to compare the dietary intake of adolescents in rural and urban settings by body mass index —these results are not shown. For adolescents from an urban setting, and between the normal weight, underweight, overweight, and obese groups, no significant difference in dietary intake was observed. For obese adolescents in a rural setting, however, more energy, carbohydrates, sugar and iron intakes were observed, when comparing the obese group with the other groups (2118.0 ± 363.7 kcal/d, 287.9 ± 50.6 g/d, 52.2 ± 24.1 g/d and 18.7 ± 5.6 mg/d, respectively).

**Table 4:** 7-d nutrient intake for adolescents age 13 y attending secondary schools by body mass index.

Macronutrient	Normal weight mean	Underweight mean	Overweight mean	Obese mean	*P*-value
	(95% CI)	(95% CI)	(95% CI)	(95% CI)	
	*n* = 460	*n* = 236	*n* = 75	n = 23	
Energy (kcal/d)	1673.6 (1642.2–1705.0)	1571.9 (1530.1–1613.7)	1742.1 (1657.7–1826.5)	1987.6 (1825.8–2149.3)	< 0.001
(MJ/d)	7.0 (6.9–7.1)	6.6 (6.4–6.8)	7.3 (6.9–7.6)	8.3 (7.6–9.0)	
Protein (g/d)	62.4 (60.9–63.8)	56.9 (55.1–58.7)	64.2 (60.4–67.9)	70.5 (62.5–78.5)	< 0.001
Carbohydrate (g/d)	231.2 (226.3–236.1)	218.5 (211.9–225.2)	235.4 (224.1–246.8)	269.7 (246.7–292.7)	< 0.001
Crude fiber (g/d)	2.9 (2.8–3.1)	2.8 (2.6–2.9)	3.0 (2.7–3.4)	4.1 (2.9–5.4)	NS
Sugar (g/d)	33.8 (32.3–35.3)	33.6 (31.6–35.6)	35.3 (30.8–39.9)	48.9 (39.0–58.7)	< 0.001
Fat (g/d)	55.7 (54.4–57.0)	52.0 (50.1–54.0)	60.6 (56.7–64.5)	70.3 (63.4–77.0)	< 0.001
Cholesterol (mg/d)	225.2 (216.5–233.8)	212.1 (200.8–223.4)	234.7 (209.9–259.5)	262.5 (209.2–315.8)	NS
Saturated fatty acid (g/d)	10.9 (10.4–11.3)	10.4 (9.8–11.0)	11.9 (10.2–13.6)	13.1 (11.6–14.6)	NS
Monounsaturated fatty acid (g/d)	8.4 (8.1–8.8)	7.8 (7.4–8.2)	8.3 (7.6–9.0)	9.9 (8.1–11.7)	NS
Polyunsaturated fatty acid (g/d)	6.1 (5.9–6.4)	5.7 (5.4–6.0)	6.3 (5.8–6.9)	7.8 (6.3–9.3)	NS
**Micronutrients**					
Vitamin D (μg/d)	0.6 (0.4–0.8)	0.5 (0.4–0.7)	0.8 (0.3–1.3)	0.4 (0.0–0.8)	NS
Sodium (mg/d)	2296.3 (2232.0–2360.6)	2168.3 (2084.0–2252.5)	2470.0 (2293.1–2646.8)	2810.4 (2411.0–3209.7)	< 0.001
Potassium (mg/d)	1051.0 (1023.4–1078.6)	955.1 (920.9–989.4)	1105.2 (1026.7–1183.6)	1243.4 (1131.2–1356.0)	NS
Calcium (mg/d)	380.7 (364.5–396.8)	355.5 (333.3–377.7)	418.0 (372.8–463.1)	404.0 (345.0–463.1)	NS
Magnesium (mg/d)	112.6 (119.3–115.9)	115.5 (105.0–126.0)	119.5 (110.5–128.5)	131.3 (115.8–146.9)	NS
Phosphorus (mg/d)	863.6 (837.1–890.1)	770.1 (741.7–798.6)	885.1 (819.9–950.4)	1017.8 (874.7–1160.8)	< 0.001
Iron (mg/d)	14.2 (13.8–14.6)	13.5 (13.0–14.1)	14.9 (13.8–16.0)	17.3 (15.1–19.6)	< 0.001

CI-Confidence interval

NS-Not significant

**Table 5:** 7-d nutrient intake for adolescents age 13-y-old attending secondary schools by places of residence.

Macronutrients	Overall mean (95% CI)	Urban Mean (95% CI)	Rural Mean (95% CI)	*P*-value
	*n* = 794	*n* = 399	*n* = 395	
Energy (kcal/d)	1659.0 (1634.6–1683.3)	1612.3 (1581.6–1643.1)	1706.1 (1668.7–1743.4)	< 0.001
(MJ/d)	6.9 (6.8–7.0)	6.8 (6.6–6.9)	7.1 (7.0–7.3)	
Protein (g/d)	61.2 (60.1–62.2)	59.3 (57.9–60.6)	63.1 (61.1–64.8)	0.001
Carbohydrate (g/d)	229.0 (225.3–232.7)	221.6 (216.8–226.3)	236.4 (230.8–242.0)	< 0.001
Crude fiber (g/d)	2.9 (2.8–3.1)	3.0 (2.8–3.1)	2.9 (2.7–3.1)	NS
Sugar (g/d)	34.3 (33.1–35.4)	34.1 (32.3–35.8)	34.5 (33.0–36.1)	NS
Fat (g/d)	55.5 (54.4–56.5)	54.6 (53.2–56.0)	56.4 (54.8–57.9)	NS
Cholesterol (mg/d)	223.3 (216.6–229.9)	202.6 (194.2–211.1)	244.1 (234.1–254.0)	< 0.001
Saturated fatty acid (g/d)	10.9 (10.5–11.2)	10.8 (10.4–11.3)	10.9 (10.4–11.5)	NS
Monounsaturated fatty acid (g/d)	8.3 (8.0–8.5)	8.3 (7.9–8.6)	8.3 (7.9–8.6)	NS
Polyunsaturated fatty acid (g/d)	6.0 (5.9–6.2)	5.9 (5.7–6.2)	6.2 (5.9–6.4)	NS
**Micronutrients**				
Vitamin D (μg/d)	0.6 (0.5–0.7)	0.7 (0.5–0.9)	0.5 (0.4–0.6)	NS
Sodium (mg/d)	2289.5 (2239.9–2339.1)	2241.9 (2177.2–2306.6)	2337.6 (2262.3–2412.9)	NS
Potassium (mg/d)	1033.2 (1012.3–1054.1)	974.8 (949.3–1000.3)	1092.2 (1059.9–1124.5)	< 0.001
Calcium (mg/d)	377.4 (365.1–389.7)	389.8 (370.8–408.8)	364.9 (349.2–380.6)	0.047
Magnesium (mg/d)	114.7 (110.9–118.5)	112.7 (107.8–117.6)	116.7 (111.0–122.4)	NS
Phosphorus (mg/d)	842.3 (823.0–861.6)	804.6 (781.0–828.2)	880.4 (850.3–910.5)	< 0.001
Iron (mg/d)	14.2 (13.8–14.5)	14.0 (13.6–14.5)	14.3 (13.9–14.8)	NS

CI- Confidence interval

NS-Not significant

In addition, no correlation was found between adolescents’ body mass index and other obesity risk factors like self-reported physical activity, SES, parental education level, and family history—these results are not shown.

## Discussion

### Dietary intake among adolescents by gender and its comparison with Malaysian RNI

Overall, males had a 10.1% higher mean energy intake compared to female adolescents. The majority of male energy intake was derived from carbohydrates, proteins, fats, and saturated fat; this finding was similar to other published studies, such as the Young Hearts Project [25], which reported that adolescent males aged 12 to 15-years consumed significantly more energy, compared to female adolescents (13.1 ± 3.58 MJ/d vs 8.4 ± 2.46 MJ/d); another study showed an increased intake of energy among adolescent males (mean, 12.9 ± 3.5 MJ/d) versus females [26]. Similar trends in total energy intake were found between boys and girls in other studies, such as the Young Hearts Project [25] and the Goteborg adolescent studies [26], yet the overall energy intake by the Malaysian adolescents participating in this study was lower than those two studies. Despite the 7-day diet history being used during the Young Hearts Project, the range of energy intake for adolescents aged 12 to 15 years was greater than the current study, which only involved adolescents aged 13-years. Older adolescents have different energy requirements compared to younger adolescents, which explains the range of energy intake. Findings on energy intake from the current study are comparable with another adolescent study conducted in Malaysia; [27] although the energy intake findings of the current study are comparable with this other study, it should be noted that we used different methods to investigate dietary intake—the Eating Behaviors Questionnaire and a three-day dietary record were used by the previous study. Because the current MyHeARTs study (2014) is an on-going longitudinal study, trends of energy and dietary intake by adolescents can be monitored.

Compared with the Malaysian RNI, the energy intake of the adolescents in current study reached an average of 65.5% (rural) and 66.3% (urban) for males, and 71.2% (rural) and 75.7% (urban) for females. Energy intake aside, male adolescents in the current study also showed higher carbohydrates, fats, cholesterol, saturated fat, MUFA and proteins intakes, compared with female adolescents (p-value <0.001). Comparing the actual macronutrient intake against the Malaysian recommended nutrient intake (RNI), we found that both male and female adolescents consumed adequate amounts of proteins, carbohydrates, fats, cholesterol and saturated fat. Even though female adolescents consumed adequate proteins, their iron intake did not fulfill RNI requirements. In line with the current study, another study in Malaysia reported similar results, whereby female adolescents only achieved 61% of their recommended iron intake [28]. Despite sufficient carbohydrates consumption, crude fiber intake among adolescents was found to be very low, and much lower than the Malaysian RNI (2.5 g/d vs 26-38g/d). This trend of inadequate macro and micronutrient intakes is similar to a cross-sectional study that observed 382 obese adolescents in the Kajang District, Selangor State; [27] in our study, both male and female adolescents showed inadequate energy and nutrient intake, including carbohydrates, fiber, vitamin D, calcium, and iron; because the focus of the study in Kajang District was only obese adolescents, the possibility of underreporting must be taken into consideration.

As for micronutrients, the overall sodium intake in our study (2289.5 ± 920.9 mg/d) exceeded the recommended intake, as necessitated by the Malaysian RNI (<2000 mg/d) [24,29]. In Malaysia, little is known of the current salt intake of adolescents. That said, a study by Moy, Gan, and Siti Zaleha (2006) and Zalilah et al. (2006) reported that ‘keropok’, which is a type of local snack that is high in salt and made from shrimp or fish mixed with flour, was a common snack consumed by adolescents [30,15]. Further investigation is needed to explore the types of foods taken by adolescents in a single meal; this is important, because it would help to educate and prevent adolescents from continuing with practices that may negatively impact their future health, practices that might aid the development of non-communicable diseases. Regardless of gender and geography, all adolescents consumed inadequate levels of calcium and vitamin D, when compared with the RNI. This finding was comparable to another study of adolescents in Malaysia [31], where similar trends were found in other countries, such as Korea [32], Brazil [33] and other European countries [34]. One study carried out in the State of Sabah, Eastern Malaysia, reported that calcium and iron intakes for adolescents aged 15 years was lowest compared to other nutrients, at 33.4% and 47.2% of the Malaysian RNI respectively [15]. In our study, calcium and vitamin D were consumed the least (< 50% of the RNI), for both males and females. These findings suggest a pro-active intervention is required if nutritional intake for adolescents to be optimized. Since 2006, Malaysia has adopted a “school milk programme” for school-aged children, yet surprisingly the uptake of milk and milk products among adolescents remains low, as was observed during this study. Since the number of foods fortified with vitamin D is minimal in Malaysia, our findings on Vitamin D intake are also not surprising.

### Comparison of adolescents’ dietary intake by BMI and places of residence

Obese adolescents in the current study had a higher energy intake, 15.8% more than normal weight adolescents—and more than 30% of this energy intake by these obese adolescents came from fats intake (70.3 g/d). Contradicting the current findings, another study reported that the crude energy intake among the overweight class of adolescents was the lowest, compared to normal and underweight adolescent classes—noting that this other study was adjusted by body weight (p-value <0.001) [17]; it must be also noted that this other study used a 3-day self-reporting food record, and thus the divergent findings of our current study might be due to the different methods used to assess dietary intake. For the current study, adolescents from rural schools had a 5.5% increase of energy intake (1706 kcal/d) compared to those from urban schools (1612 kcal/d). Such findings are similar to a study undertaken in the Chapra District in the State of Bihar in rural India, where the average food intake was higher for adolescent males from rural areas [16]. Furthermore, we found that adolescents in rural settings consumed greater amounts of high-cholesterol foods, which was similarly observed in an Ecuadorian study [35]. For the rural contingent alone, our current study showed a significant difference in energy intake—carbohydrates and sugars—between the obese group and the normal weight group (results not shown): this indicates a disparity between the dietary intake of adolescents, in urban versus rural settings; this also justifies the need for an in-depth educational programme on nutrition, especially for adolescents in rural areas, to help them improve their food preferences and intake, and to help them minimize the risks of developing non-communicable diseases later in life.

The current study did not reveal a significant difference in sugar intake between adolescents from urban and rural jurisdictions, results that were different than the Ecuadorian [35] study, which found that sugar intake was higher among urban adolescents versus their rural counterparts, with a median added-sugar intake of 86.5 g/d (approximately 17 teaspoons/d). Another study from the United States, of children and adolescents, showed that sugar-sweetened beverage topped all other food and beverage options as the primary source of sugar intake [14,36]. Comparing sugar intake with BMI, the differences we observed in our study between obese and normal weight adolescents was higher, by 15.1 g/d, and was 30.9% higher when compared to the normal weight adolescent group (*p-value* < 0.001). The 2005 Malaysian RNI guidance limits adolescent sugar consumption to < 15% of the total carbohydrates allowance, while the 2003 WHO guidance limits sugar intake for adolescents to < 10% of the total carbohydrates allowance. Thus, obese adolescents in present study consumed relatively higher amounts of sugar either from food or beverages (approximately 10 teaspoons per day). Other studies have shown that high sugar intake in overweight or obese adolescents is strongly correlated with sugar consumed via sugar-sweetened beverages [36–39]. The Malaysian Adult Nutrition Surveys (MANS) revealed that 59% of Malaysians consumed sugar and sweetened condensed milk on a daily basis [40] which would affect their child’s sugar intake. Middle-income counties should follow the practices and methods of higher-income countries, as observed in the National Health and Nutrition Examination Survey 1999–2008 [41]; that study showed a reduction of sugar intake from 100.1 g/d to 76.7 g/d, albeit sugar intakes were still greater than the recommended RNI levels. This reduction may be explained by increased public interest regarding the rise and prevalence of obesity, by a low carbohydrate diet, and by a reduction in the amount of sugar-sweetened beverages sold in schools. Although sugar intake among obese adolescents in our current study was not as high as the sugar intake found in the 1999–2008 survey [41], Malaysian adolescent sugar intake still exceeded the recommended daily sugar intake for adolescents (25–40 g/d) as recommended by the Malaysian RNI. It would be interesting to see if these trends continued in local setups, especially after the implementation of a no sugar subsidy policy instigated in Malaysia in 2013; this information, regarding sugar intake, is yet to be identified, and could be studied via the dietary assessments undertaken during the second wave of MyHeARTs from 2014.

### Strengths and limitations

This study has several strengths. Firstly, the 7-day diet history was conducted on a one-to-one basis, with each adolescent interviewed by a trained dietician. Next, the interviewers used several tools to assist their data collection: they used visual representations of household measurement tools—plate, cup, glass, bowl, ladle, spoon; they used a flip-chart that outlined common Malay, Chinese and Indian foods; they used a diet history collection tool [19,26,42]. A 7-day diet history was used because a 24-hours diet recall alone will not adequately capture the precise diet of each participant [43]; a 7-day diet history also gives a better view of emerging dietary patterns, especially variations in macronutrient intake [44]. Furthermore, when studying younger persons, it has been shown that better energy intake estimates can be obtained at the group level over than the weighed record method; the weighed record method is also more expensive than analysis at the group level; furthermore, the weighed record method is not suitable for population-based studies [44].

By using a 7-day diet history, the habitual intake of our adolescent cohort was captured [26,43,45]; most importantly, this 7-day diet history is better suited to overcoming the age-related bias that is a typical of normal food intake reporting [46]. Our study is the largest study of adolescent dietary intake in Malaysia. Dietary data obtained from our initial cohort will improve the success of future dietary planning, and will help with current interventions, undertaken by caretakers, schools and by adolescents themselves, as each works to improve the general health of our students.

The time taken to conduct each interview, approximately 30 minutes or more per adolescent, can be reduced going forward. In the future, and during the 2016 MyHeARTs study specifically, a validated food frequency questionnaire will be used to help minimize the time required to collect dietary information. Furthermore, participant recall bias is one of the main areas of error during a dietary assessment, and is a common problem of all dietary assessment research, across all ages. However, this was minimized during the current study, and can be minimized during any dietary assessment research, by using qualified and trained dietitians to conduct the interviews.

## Conclusions

There are three main findings from this baseline cohort study. First, male adolescents have higher energy and other macronutrient intakes compared to females. Second, adolescents from rural schools have higher energy and cholesterol intakes compared to adolescents from urban schools; the energy consumption of obese adolescents in rural settings was higher than their urban counterparts. Third, sugar and fat consumptions of obese adolescents in rural schools is higher compared to the normal and underweight groups of this same geographical contingent. Since MyHeARTs is a continuing longitudinal study, we did a follow-up at age 15-years in 2014, and will continue the follow-up at age 17-years in 2016. This will help determine the trend of dietary intake in early adolescence, and will assist the creation of a tailored intervention, and the creation of a robust educational tool for an effective prevention programme in Malaysia. Healthy school environments are important and a programme to make this happen is needed, to help reduce the gap of unhealthy dietary intake amongst adolescents, and to improve their health.

## Supporting Information

S1 Data MyHeARTs_raw. (SAV)
